# Genetic Variants and Soluble Isoforms of PD-1/PD-L1 as Novel Biomarkers for Pancreatic Ductal Adenocarcinoma (PDAC) Susceptibility and Prognosis

**DOI:** 10.3390/biomedicines13092246

**Published:** 2025-09-12

**Authors:** Marwa Hassan, Walaa H. El-Maadawy, Yasmine Elhusseny, Fatma Elbatol Agamy, Sally A. Fahim, Mahmoud Balata

**Affiliations:** 1 Immunology Department, Theodor Bilharz Research Institute, Giza 12411, Egypt; 2 Pharmacology Department, Theodor Bilharz Research Institute, Giza 12411, Egypt; w.elmadawy@tbri.gov.eg; 3 Medical Biochemistry and Molecular Biology Department, School of Medicine, NewGiza University, Giza 12585, Egypt; yasmine.youns@ngu.edu.eg; 4 Faculty of Medicine, Al-Azhar University, Cairo 11884, Egypt; drfatmaagamy@gmail.com; 5 Biochemistry Department, School of Pharmacy, NewGiza University, Giza 94114, Egypt; sally.atef@ngu.edu.eg; 6 University Hospital Bonn, Venusberg-Campus 1, 53127 Bonn, Germany; mbal1@uni-bonn.de; 7 University Hospital Rostock, Ernst-Heydemann-Straße 6, 18057 Rostock, Germany

**Keywords:** immune checkpoint, pancreatic ductal adenocarcinoma, PD-1, PD-L1, single nucleotide polymorphism, rs4143815, rs7421861

## Abstract

**Background:** Pancreatic ductal adenocarcinoma (PDAC) is a highly aggressive neoplasm often diagnosed at advanced stages. Immune checkpoint molecules, particularly programmed cell death protein-1 (PD-1) and its ligand PD-L1, are pivotal in tumor immune evasion. Genetic polymorphisms in PD-1/PD-L1 and their soluble isoforms (sPD-1/sPD-L1) may influence individual susceptibility to cancer and disease progression. Therefore, this study was conducted to examine the correlation between PD-1/PD-L1 gene polymorphisms, serum levels of sPD-1/sPD-L1, and their association with PDAC susceptibility, severity, and prognostication. **Methods:** This case–control study was performed with 150 PDAC patients and 150 controls. Clinical and laboratory data, including tumor markers (CA19-9 and CEA), were recorded. Allele-specific PCR was utilized to genotype PD-1 (rs6749527 and rs7421861) and PD-L1 (rs2297136, and rs4143815). sPD-1/sPD-L1 were quantified with ELISA. Mapping of the Kaplan–Meier survival curve of mutant genes was performed. **Results:** The rs7421861 AG and GG and rs4143815 GG genotypes, together with their G-alleles, were linked to increased PDAC risk and greater tumor burden. In contrast, the rs2297136 GG genotype and G-allele conferred protection against PDAC development. Serum sPD-L1 levels, rather than sPD-1, were markedly elevated in PDAC patients, progressively increased with tumor grade, and correlated with tumor markers. Also, higher PD-L1 gene expression was associated with lower overall survival. **Conclusions:** PD-1/PD-L1 genetic variants, particularly rs7421861 and rs4143815, along with sPD-L1 levels, correlate with PDAC susceptibility and disease severity. These findings endorse the prospects of integrating immune checkpoint genetic variants and soluble biomarkers for early identification, risk stratification, prognostication, and personalized therapeutic strategies in PDAC management.

## 1. Introduction

Pancreatic ductal adenocarcinoma (PDAC), the most common and aggressive pancreatic malignancy, is currently ranked as the third-leading cause of cancer-related mortality among individuals aged 50 to 79 years [1]. This disease is primarily linked to risk factors like smoking, alcohol use, and familial history, with genetic predispositions also having a significant contribution [2]. Epidemiological studies have revealed a rising incidence of PDAC, particularly among younger populations [1]. Also, the mortality rates of PDAC are increasing by ~1% each year and are anticipated to become the second most frequent cause of cancer fatalities by 2030 [3]. It often manifests with nonspecific symptoms, including abdominal pain, weight loss, and jaundice, which delays diagnosis until the cancer has progressed. Imaging modalities such as CT and MRI are essential for tumor detection, but they may miss small lesions or those with atypical features [4]. Therefore, identifying effective screening methods is vital for earlier diagnosis and improving patient outcomes [5,6].

Research into genetic factors, including single-nucleotide polymorphisms (SNPs), offers a promising avenue for exploring the relationship between genetic predisposition and cancer development [7,8]. These polymorphisms may contribute to individual variations in immune response, disease evolution, and prognosis, creating new prospects for targeted therapies and personalized treatment strategies in the ongoing battle against this serious illness [9].

**Figure 1:**
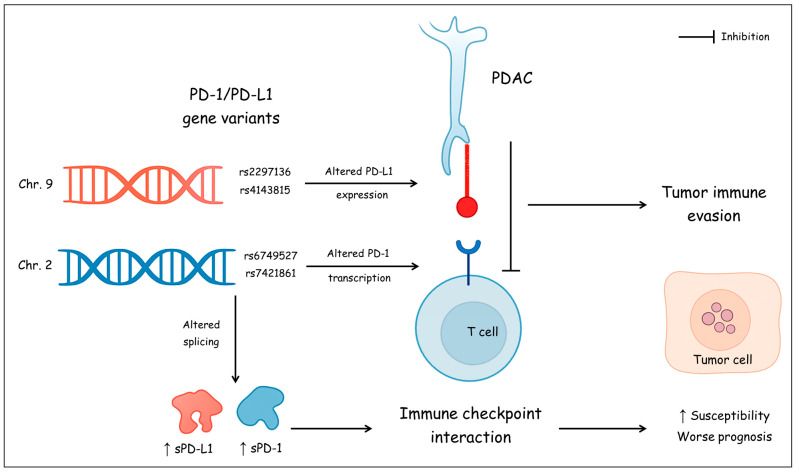
A schematic figure summarizing the proposed mechanism linking PD-1/PD-L1 variants and soluble isoforms to PDAC susceptibility and prognosis.

The programmed cell death protein-1 (PD-1) and its ligand PD-L1 are key components in regulating immune responses [10]. PD-1, a receptor expressed on the surface of T cells, interacts with PD-L1, which is abundant on tumor cells. This interaction suppresses T cell activity, enabling cancer cells to evade the immune system [11]. Variations in the PD-1 and PD-L1 genes may affect transcription, mRNA stability, or protein expression levels, thereby altering the functional activity of the PD-1/PD-L1 immune checkpoint pathway. For instance, certain polymorphisms may enhance or reduce PD-1 expression on T cells or alter PD-L1 expression on tumor cells, impacting the immune system’s capacity to attack cancer [12]. This indicates that an individual’s genetic makeup could affect the susceptibility to malignancy, aggressiveness of disease, and the patient’s response to therapy. Therefore, understanding these genetic relationships holds essential clinical ramifications. It paves the way for the development of more personalized treatment regimens, where therapies are tailored to fit the genetic profiles of patients [13]. To explore this relationship, we focused on investigating specific SNPs in PD-1 and PD-L1 genes selected for their established functional roles and associations in other malignancies. The PD-1 SNP rs7421861(intronic) has been linked to altered risk in esophageal, colorectal, hepatocellular, and lung carcinomas [14,15,16,17]. For PD-L1, we analyzed two SNPs within its 3’ untranslated region (3’UTR) of the CD274 gene on chromosome 9p24, a key regulatory region for mRNA stability and translation: rs4143815, which disrupts a binding site for the tumor suppressor miR-570, up-regulating PD-L1 expression and increasing the risk of multiple malignant disorders [18,19], and rs2297136, which has been associated with cancer prognosis and is predicted to affect miRNA binding [20]. An additional PD-1 variant, rs6749527, was included to provide broader coverage of the gene. Examining these functionally relevant polymorphisms offers a targeted approach to understanding how genetic variation in immune checkpoints influences PDAC susceptibility and progression (Figure 1).

In addition to genetic polymorphisms, soluble isoforms of PD-1 (sPD-1) and PD-L1 (sPD-L1), produced through alternative splicing or proteolytic cleavage, have recently emerged as potential modulators of antitumor immunity [21]. Elevated serum levels of sPD-1/sPD-L1 are associated with cancer progression, including non-small cell lung carcinoma (NSCLC); however, their significance in PDAC remains underexplored [22,23]. Also, persistent high expression of PD-1 in tumor-infiltrating lymphocytes is linked to unfavorable prognosis and recurrence of malignancy [24]. Accordingly, this study aimed to investigate the association between PD-1/PD-L1 gene polymorphisms (rs6749527, rs7421861, rs2297136, and rs4143815), along with serum levels of sPD-1 and sPD-L1, and the susceptibility, progression, and prognostication of PDAC. This aids in establishing reliable predictive and diagnostic tools that can allow risk stratification, optimize surveillance strategies, and anticipate tumor aggressiveness, thereby enabling timely and tailored therapeutic interventions to improve patient outcomes.

## 2. Materials and Methods

### 2.1. Study Design and Population

This case–control study involved 150 confirmed PDAC patients and 150 healthy controls matched for age and gender. Patients were recruited from the Hepato-Gastroenterology Department, Theodor Bilharz Research Institute (TBRI). Controls were selected from volunteers with no history of malignancy or chronic inflammatory disorder.

All participants received comprehensive demographic profiling and clinical evaluation. Laboratory and radiological assessments included liver function tests (AST, ALT, total bilirubin, and albumin), renal function tests (urea and creatinine), carbohydrate antigen 19-9 (CA19-9), carcinoembryonic antigen (CEA), and abdominal ultrasonography. For PDAC patients, imaging studies, including CT, MRI, and/or endoscopic ultrasound (EUS), were utilized to categorize tumor burden into three radiological grades: Grade 1: localized pancreatic mass without lymphadenopathy, Grade 2: mass with regional lymph node enlargement without vascular invasion, and Grade 3: mass with lymph node involvement and invasion of vascular, biliary, or duodenal structures.

Patients demonstrating clinical or laboratory evidence of other severe unrelated systemic illnesses were excluded from the study.

Blood samples were collected from all patients at the time of initial diagnosis, prior to the initiation of any chemo- or radiotherapy to avoid the influence of chemotherapy, radiotherapy, or immunotherapy on circulating biomarkers.

### 2.2. Genotyping of PD-1/PD-L1 Variants

Genomic DNA was extracted from whole blood utilizing the GeneJET Genomic DNA Purification Kit (Thermo Fisher Scientific, Waltham, MA, USA). Two SNPs of PD-1, rs6749527 CT and rs7421861 AG, and two SNPs of PD-L1, rs2297136 AG and rs4143815 CG, were analyzed using validated allele-specific real-time PCR protocols. The variants were chosen for their recognized relationships to disease or their functional importance. The reactions were conducted with TaqMan™ Genotyping Master Mix and SNP Genotyping Assay (40X) with VIC and FAM functioning as probes for the alleles (Thermo Fisher Scientific, USA) in accordance with the manufacturer’s guidelines. A negative control was included for every SNP. The real-time PCR cycling was run on the StepOnePlus Real-Time PCR System (Thermo Fisher Scientific, USA).

### 2.3. Measurement of sPD-1/sPD-L1

Two milliliters of whole blood were centrifuged to separate the serum, which was thereafter stored at −80 °C until analysis. Serum levels of sPD-1 and sPD-L1 were quantified in duplicate using commercially available ELISA kits (Human PD-1/PD-L1 (Soluble) ELISA, Invitrogen, Carlsbad, CA, USA), following the manufacturer’s protocols, with inter- and intra-assay CVs < 10%.

### 2.4. Retrieving Genes Correlated with PDAC

The genes relevant to PDAC were obtained from the GeneCards database (https://www.genecards.org, accessed on 21 June 2025) [25]. The GeneCards database was searched using the term “PDAC” to compile a list of genes, selecting the top 100 genes with the highest relevance scores. These scores signify the genes’ significant relevance to PDAC, implying their crucial role in the disease’s biology or potential therapeutic implications.

### 2.5. Protein–Protein Interaction

The STRING protein–protein interaction (PPI) analysis for a group of proteins related to a disease helps to understand the biological context and molecular mechanisms of that disease. Specifically, it provides functional relationships, pathway mapping, biological insight, hypothesis generation, and validation of gene/protein lists. So, the higher the actual connections between the nodes than the expected, the more significant the interactions.

The STRING PPI database [26] was used to examine the interconnectivity within the chosen target genes. The network was visualized using Cytoscape V3.10.3 [27] where the middle yellow squares represent the two genes of interest connected to their neighboring genes. Moreover, the thickness of the edges represents the strength or confidence level of the interaction between the connected proteins. Thicker edges often indicate stronger or more significant interactions. This visual representation helps to highlight the varying degrees of connectivity and the importance of interactions within the protein network.

### 2.6. Mapping of Kaplan–Meier Survival Curve of Mutant Genes and Screening of Prognostic Biomarkers

The Kaplan–Meier plotter (https://server2.kmplot.com/pancreas) [28], accessed on 20 June 2025, can evaluate the survival of PDAC patients using gene expression data. Using the Kaplan–Meier plot, we evaluated the effects of mutant genes PD-1 and PDL-1 on the prognosis of PDAC.

### 2.7. Statistical Analysis

All statistical analyses were conducted using SPSS software (v25; SPSS Inc., Chicago, IL, USA). Quantitative data were presented as mean ± standard error (SE), whilst categorical variables were summarized using frequencies and percentages. Based on the outcome of the Shapiro–Wilk normality test, normally distributed data were examined with the independent samples *t*-test. However, non-normally distributed data were assessed via the Mann–Whitney U test. The categorical variables were analyzed using the Chi-square test. The consistency of genotype distribution was evaluated using the Hardy–Weinberg equilibrium test. Odds ratios (ORs) with 95% confidence intervals (CIs) were calculated to estimate the relative risk associated with each genotype. For comparisons involving three groups of continuous variables, one-way ANOVA with LSD post hoc analysis was applied. Spearman’s correlation was used to determine associations among continuous variables. A significance threshold of *p* < 0.05 was set throughout the analyses.

## 3. Results

### 3.1. Study Population

This study included 150 PDAC patients and 150 healthy controls, with no significant differences in the mean age between groups. Similarly, the distribution of sex and smoking status did not differ significantly between groups (*p* = 0.28 and *p* = 0.30, respectively). Male participants constituted 60.7% of controls and 66.7% of the PDAC group. The prevalence of smoking was comparable in both groups (50% in controls vs. 44% in PDAC) (Table 1).

Routine laboratory investigations revealed a consistent pattern of statistically significant increases in PDAC patients in comparison to controls. Liver function markers, including AST, ALT, total bilirubin, and albumin, were markedly altered in the PDAC group. Renal function parameters were also significantly elevated, with higher urea and creatinine levels in PDAC patients. This reflects compromised hepatic and renal functions, likely caused by tumor burden and hepatobiliary obstruction. The tumor markers CA19-9 and CEA were dramatically increased in the PDAC cohort (Table 1).

Radiological classification showed that 24% of PDAC patients were diagnosed at Grade 1, 30.7% at Grade 2, and 45.3% at Grade 3, signifying that the majority of cases were identified at an advanced stage. Serum concentrations of CA19-9 and CEA were increased with cancer grade; however, these trends did not achieve statistical significance (*p* = 0.113 and *p* = 0.305, respectively) (Table 2).

### 3.2. Genotyping of PD-1/PD-L1 Variants

The analysis of genotypes for the PD-1 polymorphism rs7421861 indicated that the AA genotype was the least frequent (29.3% vs. 54.7%), whereas the AG genotype was the most prevalent (58.0% vs. 39.3%) among PDAC patients. The GG genotype was also more frequent in PDAC (12.7%) vs. controls (6.0%). At the allele level, the G-allele was significantly associated with increased PDAC risk (41.7% vs. 25.7%) (Table 3).

The genotype distribution of PD-L1 rs2297136 differed significantly between groups (*p* < 0.001). The GG genotype, observed in 19.3% of controls, was detected in only 2.0% of PDAC cases. The frequency of the AA and AG genotypes was slightly higher in PDAC (68.0%) than in controls (57.3%), but did not reach significance (*p* = 0.192 and *p* = 0.056). Allelic analysis revealed that the G-allele was underrepresented in PDAC patients (36.0%) than in controls (48.0%) (*p* = 0.003), demonstrating a protective effect (OR = 0.61, 95% CI: 0.44–0.85) (Table 4).

A significant disparity in PD-L1 rs4143815 genotypes distribution was observed between the two groups (*p* < 0.001). The GG genotype was present in 64.7% of cases versus 26.0% of controls, correlating with a 5.21-fold greater risk of developing PDAC. The CC genotype was rare in PDAC (3.3% vs. 29.3%), suggesting a strong protective effect. The G-allele was predominant in patients (80.7%) compared to controls (48.3%) (*p* < 0.001), corresponding to a high-risk association (OR = 4.46, 95% CI: 3.10–6.43) (Table 5).

The PD-1 rs6749527 C > T polymorphism showed a monomorphic distribution across the study population. All subjects, comprising both PDAC patients and healthy controls, possessed the homozygous CC genotype. Consequently, no statistical analysis could be conducted for this SNP. This complete absence of CT and TT variants may be due to a very low minor allele frequency in the studied population.

The rs7421861 exhibited significant genotypic variations across the PDAC radiological grades, with the AA genotype more frequent in Grade 1 (58.3%) than in Grades 2 (15.2%) and 3 (23.5%, *p* < 0.001), while the AG and GG genotypes escalated with disease severity (*p* < 0.001). The distribution of rs2297136 genotypes did not differ significantly among the PDAC clinical grades (*p* = 0.697). Conversely, the rs4143815 showed a strong association, with the frequency of the GG genotype rising alongside higher PDAC grades (grade 1: 41.7%, grade 2: 56.5%, grade 3: 82.4%, *p* < 0.001) (Table 2).

Analysis of tumor markers displayed no substantial correlation between rs7421861 genotypes and either CA19-9 (*p* = 0.15) or CEA levels (*p* = 0.13). Similarly, no notable variations in CA19-9 or CEA levels were perceived across the different genotypes of rs2297136 (*p* = 0.23 and *p* = 0.40, respectively).

**Figure 2:**
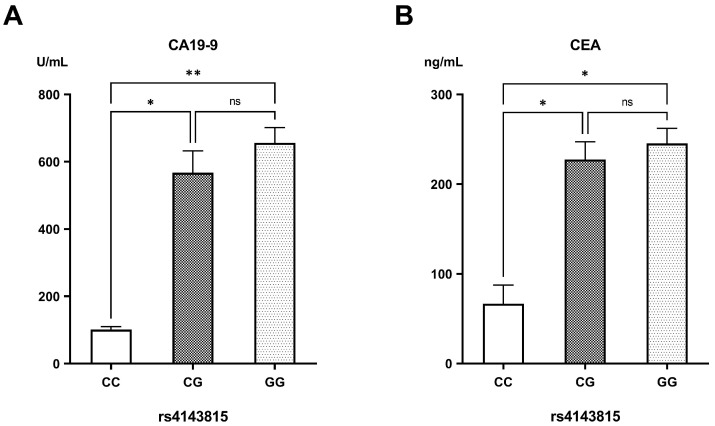
Levels of (**A**) carbohydrate antigen 19-9 (CA19-9) and (**B**) carcinoembryonic antigen (CEA) in the different genotypes of rs4143815. ns: non-significant difference; * significant difference with *p* < 0.05; ** significant difference with *p* < 0.01.

In contrast, patients carrying the GG genotype of rs4143815 had the highest CA19-9 levels (655.92 ± 45.58 U/mL), followed by those with the CG genotype (567.38 ± 64.89 U/mL) in comparison to carriers of the CC genotype (100.95 ± 9.22 U/mL) (*p* = 0.007 and *p* = 0.027, respectively). Likewise, CEA levels were markedly elevated in patients having the CG and GG genotypes (227.34 ± 19.70 ng/mL and 245.33 ± 16.82 ng/mL, respectively) compared to those with the CC genotype (66.59 ± 20.82 ng/mL) (*p* = 0.029 and *p* = 0.013, respectively) (Figure 2).

### 3.3. Measurements of sPD-1/sPD-L1

Serum analysis revealed slightly higher sPD-1 levels in PDAC patients (26.18 ± 0.53 ng/mL) than in healthy controls (25.10 ± 0.63 ng/mL), but this difference lacked statistical significance (*p* = 0.21). On the contrary, sPD-L1 concentrations were markedly increased in PDAC patients (7.85 ± 0.17 ng/mL) relative to the control group (6.40 ± 0.12 ng/mL) (*p* < 0.001).

**Figure 3:**
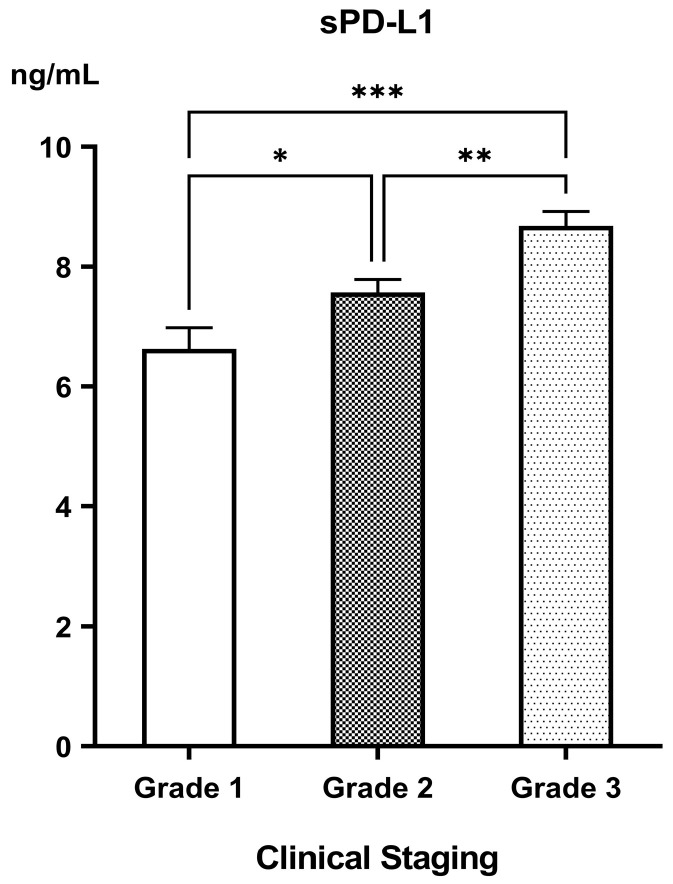
Levels of soluble PD-L1 (sPD-L1) in the different clinical gradings of PDAC. * significant difference with *p* < 0.05; ** significant difference with *p* < 0.01; *** significant difference with *p* < 0.001.

When patients were stratified by radiological grade, sPD-1 levels showed no significant variation across the grades (Grade 1: 25.26 ± 1.15; Grade 2: 26.60 ± 0.86; Grade 3: 26.38 ± 0.81 ng/mL; *p* = 0.611). Yet, sPD-L1 levels escalated consistently with the severity of disease: Grade 1 (6.63 ± 0.35 ng/mL) vs. Grade 2 (7.57 ± 0.22 ng/mL; *p* = 0.026) and Grade 3 (8.68 ± 0.24 ng/mL) (*p* < 0.001 vs. Grade 1; *p* = 0.003 vs. Grade 2) (Figure 3).

**Figure 4:**
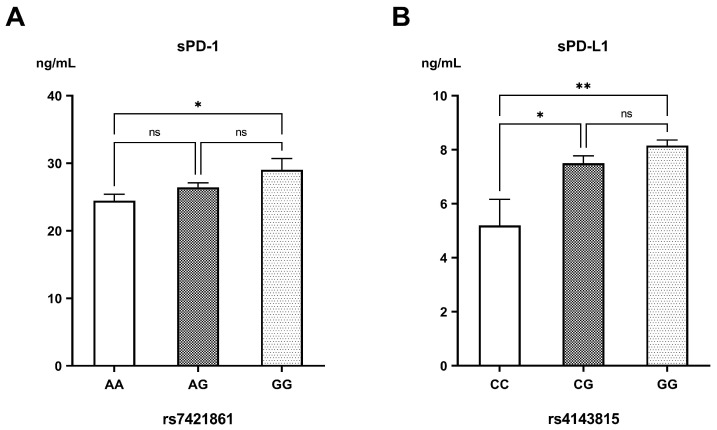
Levels of (**A**) soluble PD-1 (sPD-1) in the different genotypes of rs7421861 and (**B**) soluble PD-L1 (sPD-L1) in the different genotypes of rs4143815. ns: non-significant difference; * significant difference with *p* < 0.05; ** significant difference with *p* < 0.01.

For the genetic variants of PD-1, sPD-1 levels increased progressively with the G-allele of rs7421861 (AA: 24.46 ± 0.93 ng/mL, AG: 26.43 ± 0.67, GG: 29.03 ± 1.66 ng/mL; *p* = 0.03) (Figure 4A). Among the different genotypes of PD-L1 rs2297136, there were no significant differences in sPD-L1 concentrations (AA: 8.14 ± 0.31 ng/mL, AG: 7.71 ± 0.20 ng/mL, GG: 8.14 ± 1.67 ng/mL; *p* = 0.48). Nonetheless, sPD-L1 levels were markedly reduced in the carriers of the rs4143815 CC genotype (5.19 ± 0.97 ng/mL), compared to those having the CG (7.50 ± 0.27 ng/mL; *p* = 0.015) and GG genotypes (8.15 ± 0.20 ng/mL; *p* = 0.001) (Figure 4B).

**Figure 5:**
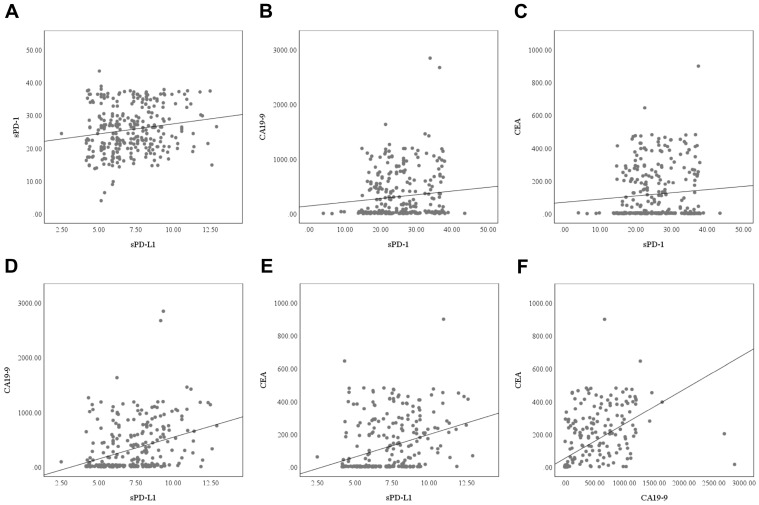
Scatterplot diagrams showing the correlations between (**A**) soluble PD-1 (sPD-1) and soluble PD-L1 (sPD-L1), (**B**) carbohydrate antigen 19-9 (CA19-9) and sPD-1, (**C**) carcinoembryonic antigen (CEA) and sPD-1, (**D**) CA19-9 and sPD-L1, (**E**) CEA and sPD-L1, (**F**) CEA and CA19-9.

Correlation analysis revealed a statistically significant albeit weak positive association between serum sPD-1 and sPD-L1 levels (r = 0.164, *p* = 0.004). While sPD-1 showed no significant correlation with CA19-9 (r = 0.111, *p* = 0.056) or CEA (r = 0.085, *p* = 0.140), sPD-L1 was significantly correlated with both tumor markers; CA19-9 (r = 0.350, *p* < 0.001) and CEA (r = 0.331, *p* < 0.001). Additionally, a robust positive correlation was identified between CA19-9 and CEA levels (r = 0.573, *p* < 0.001), indicating a potential link in their elevation in PDAC cases (Figure 5).

### 3.4. Genes Correlated with PDAC

We obtained 2149 genes correlated with PDAC. We chose the top 100 genes with the highest relevance scores. These top genes included both PD-1 and PD-L1, indicating their correlation with PDAC with high relevance.

### 3.5. Protein–Protein Interactions

**Figure 6:**
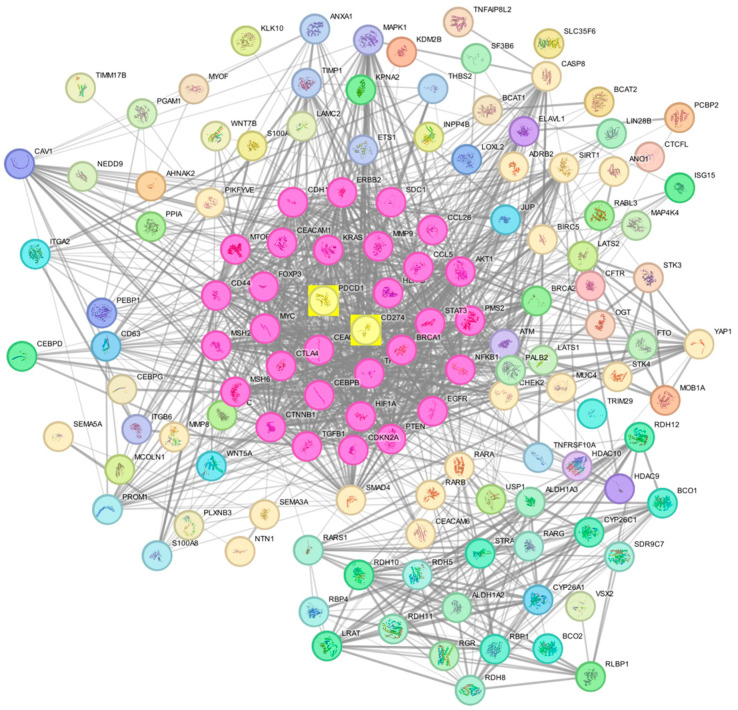
STRING Protein–protein interaction analyses. The network has 1194 edges (vs. 526 expected edges); enrichment *p*-value < 0.0001; clustering coefficient: 0.651. The thickness of the connecting edges denotes the strength of interaction. The middle yellow squares represent the two genes of interest connected to their neighboring genes.

Employing an interaction score threshold of 0.4, the STRING PPI analysis was conducted using the top 100 genes, which revealed a densely clustered network (clustering coefficient: 0.651) comprising 138 nodes connected by 1194 edges (with an anticipated number of edges being 526) (Supplementary file Table S1). This outcome suggests a significantly heightened level of interaction compared to what would be anticipated for a random set of similar size sourced from the genome (enrichment *p*-value < 0.0001, Figure 6). This indicates a high level of interaction between both PD-1 and PD-L1 and the other PDAC-related genes.

The lines connecting PD-1 and PD-L1 in the STRING network represent different types of evidence supporting their interaction, including:

(1) Co-expression: This indicates that PD-1 and PD-L1 show similar expression patterns across multiple datasets or conditions, suggesting they may functionally cooperate.
(2) Text mining: Associations are identified from published scientific literature, where PD-1 and PD-L1 are frequently mentioned together, implying a functional or biological relationship.
(3) Experimentally determined: This evidence comes from laboratory studies that directly demonstrate the interaction between PD-1 and PD-L1, such as binding assays, co-immunoprecipitation, or crystallography.
(4) Curated databases: Information is drawn from established biological databases that manually collect and verify known protein–protein interactions reported in the literature.

### 3.6. Kaplan–Meier Survival Curve Analysis of PD-1 and PD-L1 Genes and Screening of Prognostic Biomarkers

**Figure 7:**
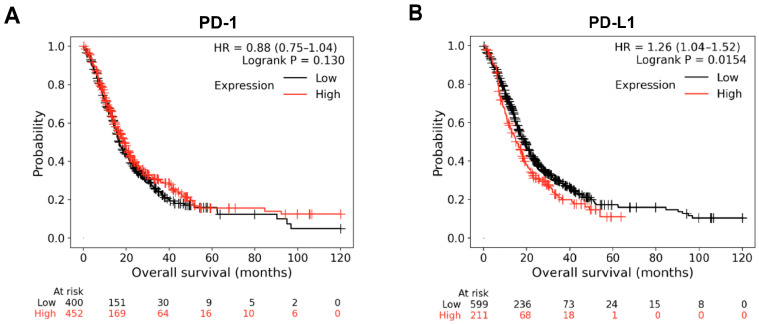
Kaplan–Meier survival curves of the (**A**) PD-1 and (**B**) PD-L1 genes.

Based on Kaplan–Meier plots, patients were divided into a high-expression group and a low-expression group according to the median expression value. The Overall Survival (OS) curves of PD-1 and PD-L1 genes were plotted. The OS curves revealed that high expression levels of PD-1 showed no significant change in OS, while high levels of PD-L1 were associated with lower OS at *p* = 0.015 (Figure 7A,B). Median survival in the high expression of PD-L1 showed 15 months of survival vs. 19.07 months in the low expression cohort.

## 4. Discussion

This study provides novel insights into the interplay between PD-1/PD-L1 polymorphisms (rs6749527, rs7421861, rs2297136, and rs4143815), their soluble isoforms’ proteins, and susceptibility to PDAC, along with their relationship to disease severity. Our data indicated that the rs7421861 AG and GG genotypes, together with the G-allele, were associated with a 2.13-, 2.27-, and 2.07-fold increase in PDAC risk, respectively, and were correlated with advanced tumor grade. On the other side, the rs2297136 GG genotype and G-allele conferred a protective effect against the development of PDAC. Individuals possessing the rs4143815 GG genotype and G-allele exhibited a greater susceptibility to develop PDAC, with odds ratios of 5.20 and 4.46, respectively. Also, they demonstrated the highest clinical grade and CA19-9/CEA levels. Furthermore, it was revealed that elevated sPD-L1 levels could serve as a potential biomarker for advanced illness. In addition, higher PD-L1 gene expression was associated with lower OS. This signifies a substantial contribution of PD-1/PD-L1 immunogenetics to the pathogenesis and advancement of PDAC.

PDAC is one of the most lethal malignancies, demonstrating a 5-year survival rate of less than 10%. Its subtle onset, rapid progression, and resistance to conventional therapy necessitate the identification of novel biomarkers to improve early detection and prognostication. Current biomarkers, including CA19-9 and CEA, lack sufficient specificity and sensitivity, particularly in the early stages of disease, reflecting a critical gap in clinical tools [29].

Immune checkpoint pathways, especially the PD-1 receptor and its ligand PD-L1, have garnered significant attention for their involvement in tumor immune evasion. SNPs in their encoding genes may modify their expression or functional interactions, consequently influencing cancer susceptibility, progression, and therapeutic response. Remarkably, rs7421861 (in the PD-1 gene), rs2297136, and rs4143815 (in the PD-L1 gene) have been implicated in several malignancies [19,24]. Notwithstanding these insights, no studies have thoroughly evaluated the interplay between soluble isoforms of PD-1/PD-L1, their genetic variants, and PDAC pathogenesis. Therefore, this work aimed to explore the association between PD-1/PD-L1 genetic polymorphisms, their soluble isoforms, and their implications on PDAC progression. Understanding these correlations allows the development of reliable predictive and diagnostic tools, thereby enabling timely and tailored therapeutic interventions to improve survival rates.

PD-1 rs7421861 A > G polymorphism locates PDCD1 intron on chromosome 2q37, where numerous regulatory components and splicing control elements may interact with it. Mutations in this region may disrupt the splice site, suppress translation, and modify the mRNA secondary structure [30]. Several studies have examined the effect of PD-1 rs7421861 A > G locus on the development of multiple cancers, revealing that the rs7421861 A > G polymorphism is linked to the diminished activation of T cells, which may impede the surveillance mechanism of the immune system; however, the results remain incoherent [14]. In the present study, it was observed that the G-allele of rs7421861 was significantly more common in PDAC. Also, the GG genotype corresponded to the highest sPD-1 concentration, implying a potential functional role in immune modulation. This may reflect altered PD-1 transcription or mRNA splicing mechanisms, consistent with literature demonstrating that the rs7421861 variants may impact the PD-1 isoform balance and subsequent immune checkpoint activation [24]. These findings corroborate those of Zang and his colleagues, who have stated that rs7421861 confers increased susceptibility to esophageal cancer, is linked to higher TNM stage, and correlates with up-regulated PD-1 expression in Chinese Han individuals [24]. Also, the rs7421861 in male patients has shown substantial correlation with the stages of lung adenocarcinoma and with LDH levels [17]. In addition, Ge et al. have noticed that the rs7421861 polymorphism increases the vulnerability to esophagogastric junction adenocarcinoma, colorectal cancer, and hepatocellular carcinoma [14,15,16]. Moreover, a meta-analysis has indicated that the rs7421861 elevates overall cancer risk [31]. The effect of the rs7421861 GG genotype in our study (OR = 2.27) coincided with its moderate effect size in other gastrointestinal cancers like esophagogastric junction adenocarcinoma (OR = 1.43) and hepatocellular carcinoma (OR = 2.05) [14,16]. Dissimilarly, no significant differences have been found in the allele frequencies of rs7421861 polymorphisms in Greek and Northwest Chinese Breast Cancer Patients [32,33]. However, the rs7421861 GG genotype was more prevalent in the luminal B subtype breast cancer patients, suggesting an association with worse clinical outcomes [32]. Yet, no relationship has been shown between the rs7421861 and gastric cardia adenocarcinoma [30]. The conflicting results may be ascribed to variations in sample size, study design, ethnic diversity, pathological types, and potential sampling bias in each study. Also, the living environment may influence cancer predisposition.

The rs2297136 SNP is situated in the 3’UTR of the PD-L1 gene, presumably influencing miRNA binding, the interaction between miRNAs and target mRNAs, and PD-L1 expression [20]. In this study, the rs2297136 GG genotype and G-allele conferred protection against PDAC development. Nonetheless, there was no correlation between the rs2297136 genetic variants and sPD-L1 concentration. This contrasted with a previous study in which the G-allele was associated with a greater risk of NSCLC and distant metastases [34]. As well, Wu et al. [35] have demonstrated that the GG genotype correlates with reduced PD-L1 protein expression and unfavorable prognosis in gastric cancer.

rs4143815 C  >  G, a common SNP and a binding site for miRNA-570 located at the 3’UTR of PD-L1, may attenuate the miRNA-mediated mRNA degradation, leading to up-regulation of PD-L1 expression [18]. A number of case–control studies have been conducted to investigate the association between rs4143815 and cancer susceptibility, but have yielded controversial results [19,36]. Multiple researchers have determined that carriers of the PD-L1 rs4143815 G-allele have an obvious elevation in the risk and aggressiveness of various malignancies compared to C-allele carriers, confirming our results. We observed that the rs4143815 GG genotype was more prevalent in PDAC patients, correlated with elevated levels of CA19-9 and CEA, and exhibited raised serum sPD-L1 levels, particularly in advanced grades. These findings align with prior research revealing that the GG genotype and G-allele cause a 3.73- and 1.85-fold heightened risk of gastric adenocarcinoma, respectively, and that the rs4143815 variants have a strong association with this tumor differentiation grade, depth of infiltration, and tumor node metastasis [37]. The GG genotype of the rs4143815 polymorphism has increased the susceptibility to develop hepatocellular carcinoma (HCC) and has been associated with higher concentrations of sPD-L1, yet the CC genotype is related to better survival [38,39]. In ovarian cancer, the GG genotype and G-allele have raised the risk by 2.3 and 1.5 times, respectively, and are correlated with histological type and differentiation grade [19]. In autoimmune disorders such as type 1 diabetes mellitus, the C-allele has diminished the risk of illness and is correlated with lower autoantibody levels [40]. In addition, the results of a meta-analysis have shown that the rs4143815 C  >  G variant is associated with heightened overall cancer susceptibility [18]. The effect size of the rs4143815 GG genotype in our PDAC group (OR = 5.21) was notably higher than that reported in other malignancies, such as gastric cancer (OR = 3.7) and ovarian cancer (OR = 2.3) [19,37]. In contrast, the GG genotype of rs4143815 lowered the incidence of breast cancer in Iranian women [41]. Nonetheless, other studies have displayed a negligible correlation between rs4143815 C  >  G and the risk of esophageal squamous cell carcinoma, NSCLC, and colorectal malignancies [34,36,42]. Collectively, these data suggest that this specific immune checkpoint variant may exert a particularly strong effect on PDAC susceptibility, potentially reflecting tissue-specific immune microenvironments or distinct antigenic drivers.

Soluble forms of PD-L1 (sPD-L1) and PD-1 (sPD-1) have been detected in peripheral blood and proven to suppress T cell activities, mediate tumor evasion, and promote its progression [21]. This study utilized ELISA and identified elevated sPD-L1 concentrations in PDAC patients, with this elevation correlating to disease aggressiveness. Moreover, Kaplan–Meier survival curve analysis revealed that increased PD-L1 gene expression was associated with lower OS. In agreement with our findings, higher sPD-L1 levels have been determined in colorectal cancer patients relative to controls, and in patients with local lymph node metastasis compared with those without such metastasis [43,44]. Also, sPD-L1 has been proposed as a prognostic biomarker for predicting recurrence and survival in many cancers, including HCC [45]. Additionally, sPD-L1 has been recognized as a marker to assess the efficacy of immunotherapy in melanoma [46], NSCLC [47], and renal cell carcinoma [48].

In contrast to PD-L1, sPD-1 levels in this current study lacked a significant difference between PDAC patients and controls. Contrary to our results, high sPD-1 was associated with an increased risk of HCC irrespective of clinical stage [49]. Furthermore, it has been demonstrated that elevated sPD-1 levels during treatment have a more favorable outcome in NSCLC [50], whereas high amounts of PD-1 in the peripheral blood impair the treatment efficacy of anti-PD-1 in melanoma [46] and reduce survival in PDAC.

Unlike most previous PDAC biomarker studies that have investigated either serum tumor markers (e.g., CA19-9 and CEA) or genetic variants in isolation, our work uniquely integrates PD-1/PD-L1 polymorphisms with their soluble protein counterparts within the same patient cohort, offering a plausible biological mechanism for the observed genetic risk. This dual-level strategy may improve early risk stratification (particularly in patients with equivocal imaging or borderline CA19-9 and CEA), refine prognostic predictions, and more effectively capture inter-patient variation than single-marker studies.

While this study offers valuable insights, its case–control approach imposes limitations, necessitating future validation through a larger cohort study. Second, only four variants of the PD-1 and PD-L1 genes were examined. Third, the quantification of sPD-1/sPD-L1 via ELISA presents certain limitations for clinical translation. Although ELISA assays are sensitive and widely used, they may be influenced by factors such as cross-reactivity with structurally related proteins, inter-assay variability, and lack of universally standardized reference ranges. Therefore, further validation using standardized assays is warranted. Fourth, the participants of this study were exclusively from the Egyptian population. Considering the probability of genetic variations among different ethnic groups, studies on other populations are required to ascertain the generalizability of our findings. Finally, long-term follow-up data regarding the survival and treatment response were unavailable, which is essential for assessing prognostic implications.

Taken together, our data suggest that the PD-1/PD-L1 genetic polymorphisms, particularly rs7421861 and rs4143815, along with serum sPD-L1, are relevant to the development, aggressiveness, and survival of PDAC. Specifically, the rs4143815 GG and rs7421861 AG and GG, as well as their G-alleles, are associated with heightened susceptibility and greater tumor burden, while sPD-L1 levels may serve as an indicator of disease severity. These findings reinforce the role of immune checkpoint dysregulation in pancreatic tumor biology and emphasize the prospects of integrating genetic variants and soluble immune markers into diagnostic, prognostic, and therapeutic frameworks.

Looking ahead, further studies are warranted to elucidate the impact of these genetic factors on treatment outcomes. More extensive and diverse research could clarify these linkages and assist in determining which individuals may benefit the most from specific immunotherapies. Ultimately, this evolving knowledge holds promise for improving survival rates and quality of life for those affected by this aggressive cancer.

## Figures and Tables

**Table 1:** The Demographic and clinical characteristics of the in PDAC patients and controls.

	Control (n = 150)	PDAC (n = 150)	*p* Value
**Age (year)**	58.38 ± 0.60	59.39 ± 0.92	0.18
**Gender**			
Males	91 (60.70%)	100 (66.70%)	0.28
Females	59 (39.30%)	50 (33.30%)	
**Smoking**			
No	75 (50.00%)	84 (56.00%)	0.30
Yes	75 (50.00%)	66 (44.00%)	
**Routine Lab.**			
ALT (IU/L)	22.78 ± 1.12	84.61 ± 9.93	<0.001
AST (IU/L)	25.61 ± 0.98	71.57 ± 5.29	<0.001
Total bilirubin (mg/dL)	0.60 ± 0.02	8.06 ± 0.47	<0.001
Albumin (g/dL)	3.91 ± 0.03	3.71 ± 0.22	<0.001
Urea (mg/dL)	26.15 ± 0.82	40.03 ± 1.91	<0.001
Creatinine (mg/dL)	0.94 ± 0.02	1.20 ± 0.04	<0.001
CA19-9 (U/mL)	18.44 ± 0.95	618.43 ± 35.83	<0.001
CEA (ng/mL)	2.75 ± 0.17	229.01 ± 12.73	<0.001
**Radiological grading**			
Grade 1		36 (24.00%)	
Grade 2		46 (30.70%)	
Grade 3		68 (45.30%)	

Quantitative data are expressed as mean ± standard error (SE). Qualitative data are expressed as frequencies (percentages). Pancreatic duct adenocarcinoma (PDAC); alanine aminotransferase (ALT); aspartate aminotransferase (AST); Carbohydrate antigen 19-9 (CA19-9); carcinoembryonic antigen (CEA).

**Table 2:** Levels of tumor biomarkers and distribution of PD-1/PDL1 genotypes across the PDAC clinical grades.

	Clinical Grading of PDAC			*p* Value
	Grade 1	Grade 2	Grade 3	
**CA19-9 (U/mL)**	508.53 ± 56.43	567.75 ± 49.37	690.28 ± 66.34	0.113
**CEA (ng/mL)**	201.72 ± 26.93	231.89 ± 20.65	251.67 ± 19.97	0.305
**PD-1 rs7421861**				
AA (reference)	21 (58.30%) ^a^	7 (15.20%) ^b^	16 (23.50%) ^b^	<0.001
AG (heterozygous)	13 (36.10%) ^a^	33 (71.70%) ^b^	41 (60.30%) ^ab^	
GG (variant)	2 (5.60%) ^a^	6 (13.00%) ^a^	11 (16.20%) ^a^	
A	55 (76.40%) ^a^	47 (51.10%) ^b^	73 (53.70%) ^b^	0.002
G	17 (23.60%) ^a^	45 (48.90%) ^b^	63 (46.30%) ^b^	
**PD-L1 rs2297136**				
AA (reference)	12 (33.30%) ^a^	11 (23.90%) ^a^	22 (32.40%) ^a^	0.697
AG (heterozygous)	24 (26.70%) ^a^	34 (73.90%) ^a^	44 (64.70%) ^a^	
GG (variant)	0 (0.00%) ^a^	1 (2.20%) ^a^	2 (2.90%) ^a^	
A	48 (66.70%) ^a^	56 (60.90%) ^a^	88 (64.70%) ^a^	0.725
G	24 (33.30%) ^a^	36 (39.10%) ^a^	48 (35.30%) ^a^	
**PD-L1 rs4143815**				
CC (reference)	4 (11.10%) ^a^	1 (2.20%) ^ab^	0 (0.00%) ^b^	<0.001
CG (heterozygous)	17 (47.20%) ^a^	19 (41.30%) ^a^	12 (17.60%) ^b^	
GG (variant)	15 (41.70%) ^a^	26 (56.50%) ^a^	56 (82.40%) ^b^	
C	25 (34.70%) ^a^	21 (22.80%) ^a^	12 (8.80%) ^b^	<0.001
G	47 (65.30%) ^a^	71 (77.20%) ^a^	124 (91.20%) ^b^	

Quantitative data are expressed as mean ± standard error (SE). Qualitative data are expressed as frequencies (percentages). Carbohydrate antigen 19-9 (CA19-9); carcinoembryonic antigen (CEA). ^b^ Significant difference compared to ^a^.

**Table 3:** Distribution of PD-1 rs7421861 (A > G) genotypes and alleles in PDAC patients vs. controls.

	Control (n = 150)	PDAC (n = 150)	*p* Value	Odds Ratio	Confidence Interval
**Genotype** **AA (reference)** **AG (heterozygous)** **GG (variant)**	82 (54.70%) 59 (39.30%) 9 (6.00%)	44 (29.30%) 87 (58.0%) 19 (12.70%)	<0.001		
**Genotype** **AA** **AG + GG**	82 (54.70%) 68 (45.30%)	44 (29.30%) 106 (70.70%)	<0.001	0.34	0.21–0.55
**Genotype** **AG** **AA + GG**	59 (39.30%) 91 (60.70%)	87 (58.0%) 63 (42.0%)	0.001	2.130	1.34–3.38
**Genotype** **GG** **AA + AG**	9 (6.0%) 141 (94.0%)	19 (12.70%) 131 (87.30%)	0.047	2.27	0.99–5.20
**Allele** **A (reference)** **G (variant)**	223 (74.30%) 77 (25.70%)	175 (58.30%) 125 (41.70%)	<0.001	0.48 2.07	0.34–0.68 1.46–2.92

The data are displayed as frequencies (percentages).

**Table 4:** Distribution of PD-L1 rs2297136 (A > G) genotypes and alleles in PDAC patients vs. controls.

	Control (n = 150)	PDAC (n = 150)	*p* Value	Odds Ratio	Confidence Interval
**Genotype** **AA (reference)** **AG (heterozygous)** **GG (variant)**	35 (23.30%) 86 (57.30%) 29 (19.30%)	45 (30.0%) 102 (68.0%) 3 (2.0%)	<0.001		
**Genotype** **AA** **AG + GG**	35 (23.30%) 115 (76.70%)	45 (30.0%) 105 (70.0%)	0.192	1.4	0.84–2.36
**Genotype** **AG** **AA + GG**	86 (57.30%) 64 (42.70%)	102 (68.0%) 48 (32.0%)	0.056	1.58	0.987–2.53
**Genotype** **GG** **AA + AG**	29 (19.30%) 121 (80.70%)	3 (2.0%) 147 (98.0%)	<0.001	0.09	0.03–0.29
**Allele** **A (reference)** **G (variant)**	156 (52.00%) 144 (48.00%)	192 (64.00%) 108 (36.00%)	0.003	1.64 0.61	1.18–2.28 0.44–0.85

The data are displayed as frequencies (percentages).

**Table 5:** Distribution of PD-L1 rs4143815 (C > G) genotypes and alleles in PDAC patients vs. controls.

	Control (n = 150)	PDAC (n = 150)	*p* Value	Odds Ratio	Confidence Interval
**Genotype** **CC (reference)** **CG (heterozygous)** **GG (variant)**	44 (29.30%) 67 (44.70%) 39 (26.00%)	5 (3.30%) 48 (32.0%) 97 (64.70%)	<0.001		
**Genotype** **CC** **CG + GG**	44 (29.30%) 106 (70.70%)	5 (3.30%) 145 (96.70%)	<0.001	0.083	0.03–0.22
**Genotype** **CG** **CC + GG**	67 (44.70%) 83 (55.30%)	48 (32.0%) 102 (68.0%)	0.024	0.58	0.36–0.93
**Genotype** **GG** **CC + CG**	39 (26.00%) 111 (74.00%)	97 (64.70%) 53 (35.30%)	<0.001	5.209	3.18–8.55
**Allele** **C (reference)** **G (variant)**	155 (51.70%) 145 (48.30%)	58 (19.30%) 242 (80.70%)	<0.001	0.22 4.46	0.16–0.32 3.10–6.43

The data are displayed as frequencies (percentages).

## Data Availability

All data generated or analyzed during this study are included in this published article.

## Supplementary Materials

The following supporting information can be downloaded at: https://www.mdpi.com/article/10.3390/biomedicines13092246/s1, Table S1: Top 100 genes with the highest relevance scores to pancreatic ductal adenocarcinoma using GeneCards Database.

## Abbreviations

The following abbreviations are used in this manuscript:

3’UTR	3′-untranslated region
CA19-9	Carbohydrate antigen 19-9
CEA	Carcinoembryonic antigen
HCC	Hepatocellular carcinoma
NSCLC	Non-small cell lung carcinoma
OR	Odds ratio
OS	Overall Survival
PD-1	Programmed cell death protein-1
PDAC	Pancreatic ductal adenocarcinoma
PD-L1	Programmed cell death protein ligand 1
SNPs	Single-nucleotide polymorphisms
