# Supplementary material for: Genetic Variants and Soluble Isoforms of PD-1/PD-L1 as Novel Biomarkers for Pancreatic Ductal Adenocarcinoma (PDAC) Susceptibility and Prognosis
Source: Biomedicines. 2025 Sep 12;13(9):2246. doi: 10.3390/biomedicines13092246 (PMC12467788; doi:10.3390/biomedicines13092246)
Supplement: Supplementary file 1 [file biomedicines-13-02246-s001.zip › biomedicines-3825862-supplementary.pdf]

Table S1. Top 100 genes with the highest relevance scores to pancreatic ductal adenocarcinoma using GeneCards Database.

Gene Symbol	Description	Uniprot ID	Gifts	GC Id	Relevance score
STRA6	Signaling Receptor And Transporter Of Retinol STRA6	Q9BX79	53	GC15M074179	6.981875
RARB	Retinoic Acid Receptor Beta	P10826	62	GC03P027315	6.757993
WNT7B	Wnt Family Member 7B	P56706	55	GC22M045920	6.609201
RABL3	RAB, Member Of RAS Oncogene Family Like 3	Q5HYI8	47	GC03M120686	5.776208
SLC35F6	Solute Carrier Family 35 Member F6	Q8N357	45	GC02P026764	4.539874
BRCA2	BRCA2 DNA Repair Associated	P51587	61	GC13P032315	3.763754
SMAD4	SMAD Family Member 4	Q13485	65	GC18P051028	3.740105
ATM	ATM Serine/Threonine Kinase	Q13315	66	GC11P108222	3.347945
KRAS	KRAS Proto-Oncogene, GTPase	P01116	66	GC12M035956	3.249641
CD274	CD274 Molecule	Q9NZQ7	58	GC09P005450	2.996584
TP53	Tumor Protein P53	P04637	66	GC17M007661	2.864123
C11orf65	Chromosome 11 Open Reading Frame 65	Q8NCR3	42	GC11M108308	2.862759
TGFB1	Transforming Growth Factor Beta 1	P01137	65	GC19M041301	2.562078
HIF1A	Hypoxia Inducible Factor 1 Subunit Alpha	Q16665	61	GC14P061695	2.420273
MSH6	MutS Homolog 6	P52701	61	GC02P047695	2.271903
MYC	MYC Proto-Oncogene, BHLH Transcription Factor	P01106	65	GC08P127735	2.190367
TRIM29	Tripartite Motif Containing 29	Q14134	51	GC11M120111	2.096264
BRCA1	BRCA1 DNA Repair Associated	P38398	63	GC17M043044	2.081216
MMP9	Matrix Metallopeptidase 9	P14780	66	GC20P046008	2.068706
AKT1	AKT Serine/Threonine Kinase 1	P31749	66	GC14M104769	2.040179
MIR494	MicroRNA 494		26	GC14P121384	2.035081
MIR98	MicroRNA 98		27	GC0XM053998	2.032418
MIR216A	MicroRNA 216a		28	GC02M055988	2.020654
CDKN2A	Cyclin Dependent Kinase	Q8N726	64	GC09M021967	1.984361

	Inhibitor 2A				
MIR141	MicroRNA 141		30	GC12P074085	1.969931
MTOR	Mechanistic Target Of Rapamycin Kinase	P42345	68	GC01M011106	1.95942
HDAC10	Histone Deacetylase 10	Q969S8	56	GC22M050245	1.948408
SOD2-OT1	SOD2 Overlapping Transcript 1		20	GC06M159772	1.914509
FOXP3	Forkhead Box P3	Q9BZS1	59	GC0XM049250	1.870091
TIMP1	TIMP Metallopeptidase Inhibitor 1	P01033	57	GC0XP060812	1.857497
CCL5	C-C Motif Chemokine Ligand 5	P13501	56	GC17M035871	1.846059
MIR100	MicroRNA 100		30	GC11M122152	1.833743
MIR100HG	Mir-100-Let-7a-2-Mir-125b-1 Cluster Host Gene		26	GC11M140705	1.826937
LINC00491	Long Intergenic Non-Protein Coding RNA 491		20	GC05M102582	1.826937
CFTR	CF Transmembrane Conductance Regulator	P13569	66	GC07P117287	1.821212
MIR21	MicroRNA 21		33	GC17P059841	1.819681
MIR483	MicroRNA 483		30	GC11M015126	1.808456
NFKB1	Nuclear Factor Kappa B Subunit 1	P19838	66	GC04P102501	1.779392
CDH1	Cadherin 1	P12830	62	GC16P068737	1.770331
SIRT1	Sirtuin 1	Q96EB6	62	GC10P067884	1.754899
LINC01133	Long Intergenic Non-Protein Coding RNA 1133		22	GC01P176940	1.751953
CERNA3	Competing Endogenous LncRNA 3 For MiR-645		21	GC08P056444	1.742632
BIRC5	Baculoviral IAP Repeat Containing 5	O15392	58	GC17P078214	1.740795
PVT1	Pvt1 Oncogene		33	GC08P128394	1.73406
MIR10B	MicroRNA 10b		31	GC02P176150	1.7044
H19	H19 Imprinted Maternally Expressed Transcript		37	GC11M001995	1.676329
MIR23A	MicroRNA 23a		30	GC19M108248	1.674645
PTEN	Phosphatase And Tensin Homolog	P60484	65	GC10P124078	1.664503
MUC4	Mucin 4, Cell Surface Associated	Q99102	52	GC03M195746	1.664503
MIR381	MicroRNA 381		27	GC14P121372	1.654241
SNHG14	Small Nucleolar RNA Host Gene 14		25	GC15P192315	1.600998
ERBB2	Erb-B2 Receptor Tyrosine	P04626	68	GC17P039687	1.578698

	Kinase 2				
CEBPD	CCAAT Enhancer Binding Protein Delta	P49716	52	GC08M047765	1.576732
MIR373	MicroRNA 373		29	GC19P153085	1.567309
MIR93	MicroRNA 93		30	GC07M106714	1.555751
STAT3	Signal Transducer And Activator Of Transcription 3	P40763	67	GC17M042313	1.554448
CAV1	Caveolin 1	Q03135	61	GC07P116524	1.544017
TNFRSF10A	TNF Receptor Superfamily Member 10a	O00220	57	GC08M023190	1.544017
ANXA10	Annexin A10	Q9UJ72	45	GC04P168081	1.519986
MIR30E	MicroRNA 30e		31	GC01P040754	1.519986
CTLA4	Cytotoxic T-Lymphocyte Associated Protein 4	P16410	61	GC02P204860	1.507669
SDC1	Syndecan 1	P18827	55	GC02M020200	1.507669
MIR29C	MicroRNA 29c		27	GC01M207838	1.507669
THBS2	Thrombospondin 2	P35442	60	GC06M169215	1.495139
S100A9	S100 Calcium Binding Protein A9	P06702	56	GC01P153357	1.495139
TRA-TGC7-1	TRNA-Ala (Anticodon TGC) 7-1		14	GC06M106311	1.495139
PROM1	Prominin 1	O43490	60	GC04M015965	1.482382
MIR185	MicroRNA 185		30	GC22P091538	1.482382
MIR23B	MicroRNA 23b		29	GC09P095085	1.482382
MIRLET7A2	MicroRNA Let-7a-2		25	GC11M122146	1.482382
NTN1	Netrin 1	O95631	58	GC17P153828	1.469387
MIR142	MicroRNA 142		30	GC17M058331	1.469387
UCA1	Urothelial Cancer Associated 1		30	GC19P160848	1.469387
PDCD1	Programmed Cell Death 1	Q15116	60	GC02M241849	1.45614
MIR301B	MicroRNA 301b		18	GC22P091622	1.45614
HULC	Hepatocellular Carcinoma Up-Regulated Long Non-Coding RNA		29	GC06P009612	1.453284
YAP1	Yes1 Associated Transcriptional Regulator	P46937	61	GC11P102110	1.447113
MIR486-1	MicroRNA 486-1		28	GC08M041660	1.442625
PALB2	Partner And Localizer Of BRCA2	Q86YC2	57	GC16M023603	1.434691
ANXA1	Annexin A1	P04083	60	GC09P073151	1.428825
MIR106B	MicroRNA 106b		30	GC07M106713	1.428825
CEACAM5	CEA Cell Adhesion Molecule 5	P06731	56	GC19P152590	1.414722

MIR186	MicroRNA 186		28	GC01M071067	1.414722
CD63	CD63 Molecule	P08962	56	GC12M060362	1.400294
MIR19A	MicroRNA 19a		28	GC13P091748	1.400294
MIR200A	MicroRNA 200a		29	GC01P074920	1.385519
MIR144	MicroRNA 144		26	GC17M096703	1.385519
ETS1	ETS Proto-Oncogene 1, Transcription Factor	P14921	61	GC11M128458	1.37037
LOXL2	Lysyl Oxidase Like 2	Q9Y4K0	58	GC08M023296	1.354816
S100A8	S100 Calcium Binding Protein A8	P05109	55	GC01M167351	1.354816
S100A16	S100 Calcium Binding Protein A16	Q96FQ6	44	GC01M153606	1.354816
MIR214	MicroRNA 214		31	GC01M172234	1.354816
MIR382	MicroRNA 382		25	GC14P121374	1.354816
MIR637	MicroRNA 637		22	GC19M003961	1.354816
STK4	Serine/Threonine Kinase 4	Q13043	61	GC20P044966	1.338824
ELAVL1	ELAV Like RNA Binding Protein 1	Q15717	54	GC19M007958	1.338824
MIR378A	MicroRNA 378a		31	GC05P149732	1.338824
MIR17	MicroRNA 17		28	GC13P091350	1.338824
MMP8	Matrix Metallopeptidase 8	P22894	60	GC11M140389	1.322354
PEBP1	Phosphatidylethanolamine Binding Protein 1	P30086	58	GC12P118189	1.322354
